# miR‐499 released during myocardial infarction causes endothelial injury by targeting α7‐nAchR

**DOI:** 10.1111/jcmm.14474

**Published:** 2019-07-03

**Authors:** Rui Zhou, Wenjun Huang, Xinrong Fan, Feng Liu, Liangqin Luo, Haiyang Yuan, Yu Jiang, Haiying Xiao, Zhichao Zhou, Chenliang Deng, Xitong Dang

**Affiliations:** ^1^ The Key Laboratory of Medical Electrophysiology of Ministry of Education and Medical Electrophysiological Key Laboratory of Sichuan Province, Collaborative Innovation Center for Prevention and Treatment of Cardiovascular Disease of Sichuan Province, Institute of Cardiovascular Research Southwest Medical University Stockholm Sweden; ^2^ Department of Cardiovascular Medicine The 1st Affiliated Hospital of Southwest Medical University, Southwest Medical University Stockholm Sweden; ^3^ Department of Cardiovascular Surgery The 1st Affiliated Hospital of Southwest Medical University, Southwest Medical University Stockholm Sweden; ^4^ Division of Cardiology, Department of Medicine, Heart and Vascular Theme Karolinska University Hospital, Karolinska Institute Stockholm Sweden; ^5^ Department of Plastic Surgery Shanghai 6^th^ People’s Hospital, Shanghai Jiaotong University School of Medicine Shanghai China

**Keywords:** acute myocardial infarction, endothelial injury, miR‐499, α7‐nAchR

## Abstract

The surged systemic vascular inflammation after acute myocardial infarction (AMI) aggravates the atherosclerotic endothelial injury. To explore roles of miR‐499 released from cardiomyocytes during AMI in endothelial injury. Using qPCR and ELISA, we discovered that patients with AMI had significantly increased plasma miR‐499, which was directly correlated with serum thrombomodulin, a marker for endothelial injury. Plasma of AMI patients, when incubated with human umbilical vein endothelial cells (HUVECs), significantly increased the expression of endothelial injury markers, which could be abrogated by antagomiR‐499. In vitro, neonatal rat cardiomyocytes subjected to hypoxia/reoxygenation (HX/R) released miR‐499 that could be internalized into rat pulmonary microvascular endothelial cells (RPMECs), worsening the high glucose‐induced injury. In silico analysis demonstrated that CHRNA7 encoding α7‐nAchR is a target of miR‐499, which was validated in cell lines expressing endogenous α7‐nAchR. In high glucose‐induced RPMECs injury model, miR‐499 aggravated, whereas forced CHRNA7 expression ameliorated the injury. Moreover, the perfusate from Langendorff perfused rat heart subjected to HX/R contained higher level of miR‐499 that significantly impaired the Bradykinin‐mediated endothelium‐dependent relaxation in both conduit and resistance arteries, which could be partially abrogated by antagomiR‐499. Finally, the correlation between plasma miR‐499 and endothelial injury was further confirmed in another cohort of AMI patients. We conclude that miR‐499 released from injured cardiomyocytes contributes to the endothelial injury by targeting α7‐nAchR. This study implies that miR‐499 may serve as a potential target for the treatment of the surged vascular inflammation post‐AMI.

## 1 INTRODUCTION

The cross‐talk between cardiomyocytes (CMs) and endothelial cells (ECs) is essential for the development, growth and homoeostasis of cardiovascular systems. CMs are physically interacting with coronary artery ECs and constantly communicating with distant vascular ECs through auto‐, para‐ or exo‐crine mechanisms, which guarantees a prompt responsiveness appropriate to the insults experienced by CMs. Accumulating literatures have shown that many EC‐derived molecules such as nitric oxide, endothelin‐1 and prostaglandins modulate the functions of CMs.^1^ However, reports that molecules derived from CMs, particularly under acute stress such as acute myocardial infarction (AMI), interact with ECs remain scarce.

AMI is myocardial necrosis resulting from acute obstruction of an atherosclerotic coronary artery. Immediately after AMI, there is a surged systemic vascular inflammation including endothelium injury, which promotes recurrence of a second event.^2^, ^3^, ^4^ The molecular mechanisms driving the exaggerated post‐AMI vascular inflammation are complex and begin to be elucidated. During AMI, the heighted sympathetic nerve tone causes increased mobilization of haematopoietic stem/progenitor cells into peripheral blood, which provides a pool of inflammatory cells that are recruited to the growing arterial lesions.^4^, ^5^ The increased sympathetic tone also increases the expression of cell surface adhesion molecules of ECs, attracting more leukocytes.^3^ In addition, infarcted myocardium releases reactive oxygen species (ROS), danger associated molecular patterns (DAMPs), intracellular molecules not commonly exposed to immune system, interleukin‐1ß and heat shock proteins into blood, which elicit a systemic immune response including activation of endothelial cells.^6^, ^7^, ^8^ Lastly, AMI causes the release of other cytosolic signalling molecules from CMs, for example non‐coding RNAs that upon internalization into recipient cells activate the downstream pro‐inflammatory signalling pathway.^9^

Recently, microRNAs (miR) have been shown to play critical roles in the physiology of cardiovascular system and have been implicated in the pathogenesis of cardiovascular diseases including AMI and post–AMI.^10^ For example, miR‐499 abundantly expressed in quiescent CMs is released during AMI. Therefore, it was proposed as a biomarker for the severity and extent of cardiac injury and for the prognosis.^11^ In addition, plasma miR‐499 has been shown to exert distinct functions depending on recipient cell types. In one report, miR‐499 protected CMs from apoptosis‐induced by ischaemia/reperfusion (I/R) via targeting programmed cell death 4 (PDCD4),^12^ calcineurin and dynamin related protein‐1.^13^ In other reports, plasma miR‐499 was shown to promote endothelial inflammation through targeting PDCD4, which de‐inhibited the downstream NF‐kB/tumor necrosis factor (TNF)α signalling pathway.^14^

α7 nicotinic acetylcholine receptor (α7‐nAchR), encoded by CHRNA7, is not only expressed in central and peripheral nerve systems but also on macrophages, epithelial and endothelial cells.^15^, ^16^ It is a critical regulator of the newly‐discovered cholinergic anti‐inflammation pathway (CAIP) that inhibits pro‐inflammatory cytokine release from tissue macrophages, dampening the original peripheral inflammation and protecting organs from I/R injury.^15^, ^17^, ^18^, ^19^, ^20^ Here we provide evidence that plasma miR‐499 released from injured CMs during AMI can be internalized into ECs, which contributes to the surged endothelial inflammation post‐AMI through targeting α7‐nAchR.

## 2 MATERIALS AND METHODS

### 2.1 Patients and animals

Study protocols were reviewed and approved by the ethical committee of Southwest Medical University. The Guide for the Care and Use of Laboratory Animals by the US National Institutes of Health was followed for all animal studies. Human specimens were harvested from healthy subjects or patients admitted to the Affiliated Hospital of Southwest Medical University. A written informed consent was obtained from each subject prior to the specimen collection. The procedure was conducted according the principles outlined by the declaration of Helsinki and was approved by Southwest Medical University.

A total of nine patients with AMI were recruited from the Affiliated Hospital of Southwest Medical University in 2018. AMI was diagnosed by patients’ history, symptoms, blood test and angiogram. Of the nine patients, blood samples were collected from six patients at day 5, and the other 3 were passed away. Five healthy volunteers were recruited as healthy control. The basal characteristics of subjects are summarized in Table S2.

A total of 27 patients with type 2 diabetes mellitus (T2DM) were recruited from the Affiliated Hospital of Southwest Medical University during 2016‐2017. T2DM was defined as fasting blood glucose exceeding 7 mM on two different occasions, blood glucose exceeding 11 mM two hours after oral administration of 75 g of glucose, glycated haemoglobin above 52 mM, or patients having a medical history of T2DM. Of the 27 patients, 15 were diagnosed with AMI (T2DM/MI) and the rest had no‐known complications. The basal characteristics of patients are summarized in Table S3.

Harvest of animal organs was performed in accordance with the European regulations for animal care and handling (2010/63/EU). For procurement of aorta or mesenteric artery, adult rats were anaesthetized with pentobarbital sodium (50 mg/kg *ip*) and then euthanized by cervical dislocation. For heart procurement, neonatal rats were pre‐anaesthetized in a closed chamber with 5% isoflurane in oxygen and then euthanized by cervical dislocation.

### 2.2 Isolation of neonatal rat cardiomyocytes (NRCMs)

NRCMs were isolated using a modified two‐enzyme digestion method.^21^ Briefly, whole heart was harvested from 1 to 3 days old neonatal Sprague‐Dawley (SD) rats. The hearts were rinsed in sterile PBS and minced into small pieces in a petri‐dish. The minced tissue was then digested with 0.25% trypsin at 37°C for overnight. The digestion was spun down, washed and subjected to the second digestion (0.1% typeⅡ collagenase and 1% BSA) at 37°C water bath for 5 minutes with gentle agitation. Digestion was terminated by adding 1 mL of 10% DMEM. After viability test, cells were seeded into appropriate plates based on experiment need.

### 2.3 RNA isolation and qPCR

Total RNA was isolated using Trizol method (Invitrogen). One μg of total RNA was reversely transcribed in a total volume of 10 μL with ReverTra Ace qPCR RT Master Mix kit (TOYOBO) following manufacturer's instructions. The cDNA was diluted three times, and 1 μL was used for real‐time PCR in a 20 μL reaction using SYBR Green Real Time PCR Mix (Qiagen). The PCR conditions were 95°C for 2 minutes, followed by 40 cycles of 95°C for 20’’ and 60°C for 10’’. All primers were listed in Table S1. The PCR efficiency for each primer pair was between 95% and 105%. The expression of target gene was normalized to that of GAPDH and calculated using 2^−∆∆Ct^ method as described in BioRad's qPCR manual.

### 2.4 miRNA isolation and qPCR

For miRNA analysis, total RNA from plasma, CdM or perfusate was extracted using miRNeasy Plasma Kit (Qiagen) per vendor's instructions. Two hundred and fifty nanograms of total RNA was reversely transcribed in 10 μL reaction using RevertAid First Strand cDNA Synthesis Kit (Thermo Fisher) and miR‐499‐, U6‐, or miR‐103a‐specific RT primer. The cDNA was diluted 2×, and 2 μL was used for qPCR in a 20 μL reaction using SYBR Green Real Time PCR Mix (Qiagen). The PCR conditions were the same as aforementioned. All primers were listed in Table S1. The PCR efficiency for each primer pair was between 95% and 105%. The expression of miRNA, normalized to that of U6 for cell lines and to that of miR‐103a for plasma, CdM and perfusate, was calculated using 2^−∆∆Ct^ method.

### 2.5 Western blot

Western blot was performed following the protocol reported previously.^22^ Briefly, cells were lysed using SDS lysis buffer supplemented with proteinase inhibitors, sonicated and boiled for 10 minutes. Lysates were spun at top speed for 1 minute at room temperature and supernatants were quantitated. Twenty μg of lysate was resolved with 10% SDS‐PAGE, and transferred onto PVDF membrane (Millipore, USA). The membrane was blocked with 5% milk in TBST for 1 hour, probed with anti‐α7‐nAChR antibody for overnight at 4°C, washed, and followed by secondary antibody incubation for 1 hour. The membrane was then developed with Superglow ECL (Shenggong, Shanghai), image was acquired and relative expression normalized to that of glyceraldehyde‐3‐phosphate dehydrogenase (GAPDH) was analysed by densitometry using the Molecular Analyst software (Imaging Densitometer, Bio‐Rad).

### 2.6 Enzyme linked immunosorbent assay (ELISA)

One hundred microlitres of CdM or plasma was used for analysing, IL‐1α, IL‐6, intercellular adhesion molecule 1 (ICAM‐1), monocyte chemoattractant protein 1 (MCP‐1), thrombomodulin (TM) using sandwich‐type ELISA kits (Boster Biotech., China). Briefly, target protein was captured by pre‐coated monoclonal antibody in 96‐well plate. A biotin‐conjugated detection antibody was used to bind the captured target molecules, and avidin conjugated HRP was used to amplify the signals. A microplate reader (Synergy2, BioTek) was employed to detect the optical density (OD) at 450 nm. The concentrations of target protein were calculated using the standard curve.

### 2.7 Preparation of rat heart and I/R by Langendorff

The male SD rats (250‐280 g) were anaesthetized by intraperitoneal (*ip*) injection of pentobarbital sodium (50 mg/kg *ip*). To prevent coagulation, 250 U/kg of heparin were administered intraperitoneally to the rats. Thoracic surgery was performed to remove the hearts that were immediately immersed in ice‐cold Krebs‐Henseleit (KH) buffer 23, 24 supplemented with 95% O_2_ and 5% CO_2_. The heart was mounted on Langendorff's apparatus and equilibrated with a gas mixture bubbled with 95% O_2_, 5% CO_2_ at 37°C. The whole procedure was finished in 2 minutes. To simulate I/R, the perfusion was stopped for 30 minutes and then continued for one hour. During the reperfusion, the perfusate was collected to test its effect on artery relaxation in vitro. Immediately after experiment, the heart was removed, frozen (−20°C, 30 min) and then sliced into 1mm sections perpendicularly along the long axis from apex to base. The slices were incubated in 1% triphenyltetrazolium chloride (TTC, Biodee, Beijing) in pH 7.4 buffer at 37°C for 15 minutes, fixed in 10% formaldehyde solution to confirm the success of I/R

### 2.8 Preparation of arterial rings for wire myograph studies and experimental protocols

Rats were anaesthetized with pentobarbital sodium (50 mg/kg *ip*) followed by removal of aortas and mesenteric arteries. The vessels were cleaned by removing fat and connective tissues, and subsequently cut transversely into 2 mm rings. The vessel rings were incubated with the perfusate collected from the Langendorff I/R experiment at 37℃ with 5% CO_2 _for 18 hours. Control arteries were incubated with KH buffer or the perfusate during the equilibration (control). Subsequently the incubated vessels were thoroughly washed and mounted in wire myograph (Danish Myo Technology) in the organ bath containing 6 mL KH buffer. The KH buffer was maintained at 37°C and aerated with 95% O_2_/5% CO_2_. Changes in contractile force were recorded with a Harvard isometric transducer. Following 30 minutes stabilization, the internal diameter was set at a tension equivalent to 0.9 times the estimated diameter at 100 mm Hg effective transmural pressure for rat aortic rings, while resting tension was gradually increased to 1 mN for mesenteric arteries. At the end of the equilibration, the vessels were exposed to KCl twice (50 and 100 mM, consequentially) to check the contractility.^24^ Thereafter, vessels were allowed to equilibrate in fresh KH buffer for 30 minutes before initiating different experimental protocols. Phenylephrine (PE) was applied to reach the stable pre‐constriction. Vessels with unstable pre‐constriction were excluded from the study. Subsequently, bradykinin concentration responses (10^−10^‐10^−5^M) were conducted to evaluate endothelium‐dependent relaxation (EDR).

### 2.9 Statistical analysis

Two groups comparison was performed using paired or non‐paired Student's *t* test. Plasma data were calculated with Mann‐Whitney *U* test and Wilcoxon test. Concentration‐response curves were analysed with 2‐way analysis of variance with repeated measurement. Spearman correlation test was used to analyse correlations between miR‐499 and TM. Except data regarding Concentration‐response curves (mean ± standard error of the mean [SEM]), all the other experimental data are presented as mean ± standard deviation (SD). Statistical significance was accepted when *P* < 0.05.

## 3 RESULTS

### 3.1 Patient characteristics

Basal characteristics of patients with AMI and their age‐matched controls are shown in Table S2. The patients with AMI had higher body mass index, total cholesterol, High Density Lipoprotein (HDL), Low Density Lipoprotein (LDL), high sensitivity troponin (hs‐TNT), myoglobin (MYO) and creatine kinase‐MB (CK‐MB) compared to the healthy controls. None of the healthy controls was on any medications. Basal characteristics of patients with T2DM with or without AMI are listed in Table S3. The patients with AMI had higher levels of Hemoglobin, LDL, hs‐TNT, MYO and CK‐MB compared to those without AMI.

### 3.2 Plasma miR‐499 level is increased in patients with AMI, which is directly correlated with endothelial injury

**Figure 1:**
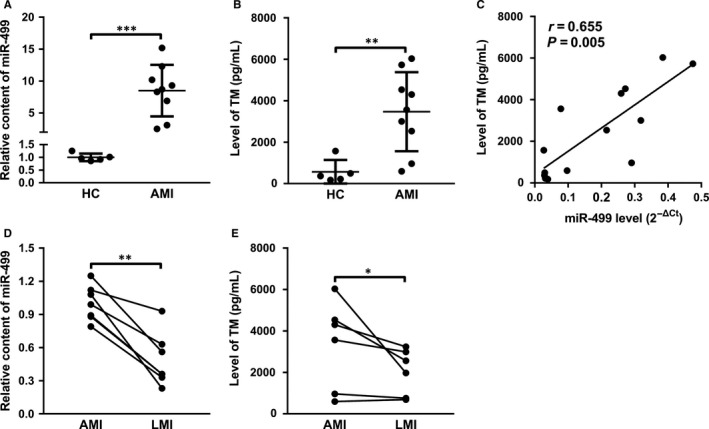
Increased levels of plasma miR‐499 after AMI are directly correlated with endothelial damage. A, Plasma miR‐499 is ~7‐fold higher in patients with AMI (n = 9) than HC (n = 5) (*P* < 0.001). B, Plasma TM is significantly higher in AMI (n = 9) than HC (n = 5) (*P* < 0.01). C, Plasma miR‐499 was positively correlated with the TM in the patients with or without AMI (r = 0.655, *P* = 0.005). D, Plasma miR‐499 is significantly higher in AMI than LMI (n = 6 in each and *P* < 0.01). E, Plasma TM is significantly higher in AMI than LMI (n = 6 in each and *P* < 0.05). The levels of miR‐499 were normalized to that of U6 RNA. Data are presented as ‘Mean ± SD’. Mann‐Whitney *U* test (A, B), Spearman correlation test (C) and Wilcoxon test (D, E) were used for statistical analysis, with * denoting *P* < 0.05, ** *P* < 0.01, and *** *P* < 0.001

Plasma miR‐499 has been proposed as a biomarker for AMI that is usually accompanied by a severe systemic inflammation and endothelial dysfunction.^11^ Indeed, plasma miR‐499 was 7‐fold higher in patients with AMI than HC (Figure 1A). Interestingly, plasma TM, a marker for microvascular endothelial cell damage,^25^ was significantly elevated (Figure 1B) and directly correlated with plasma miR‐499 in patients with AMI (Figure 1C). Of note, the levels of both plasma miR‐499 (Figure 1D) and TM (Figure 1E) were significantly higher on day 1 than day 5 (late stage of AMI, LMI) post‐AMI. These results suggest a potential cross‐talk between myocardium and endothelium.

### 3.3 CHRNA7 is a target of miR‐499

**Figure 2:**
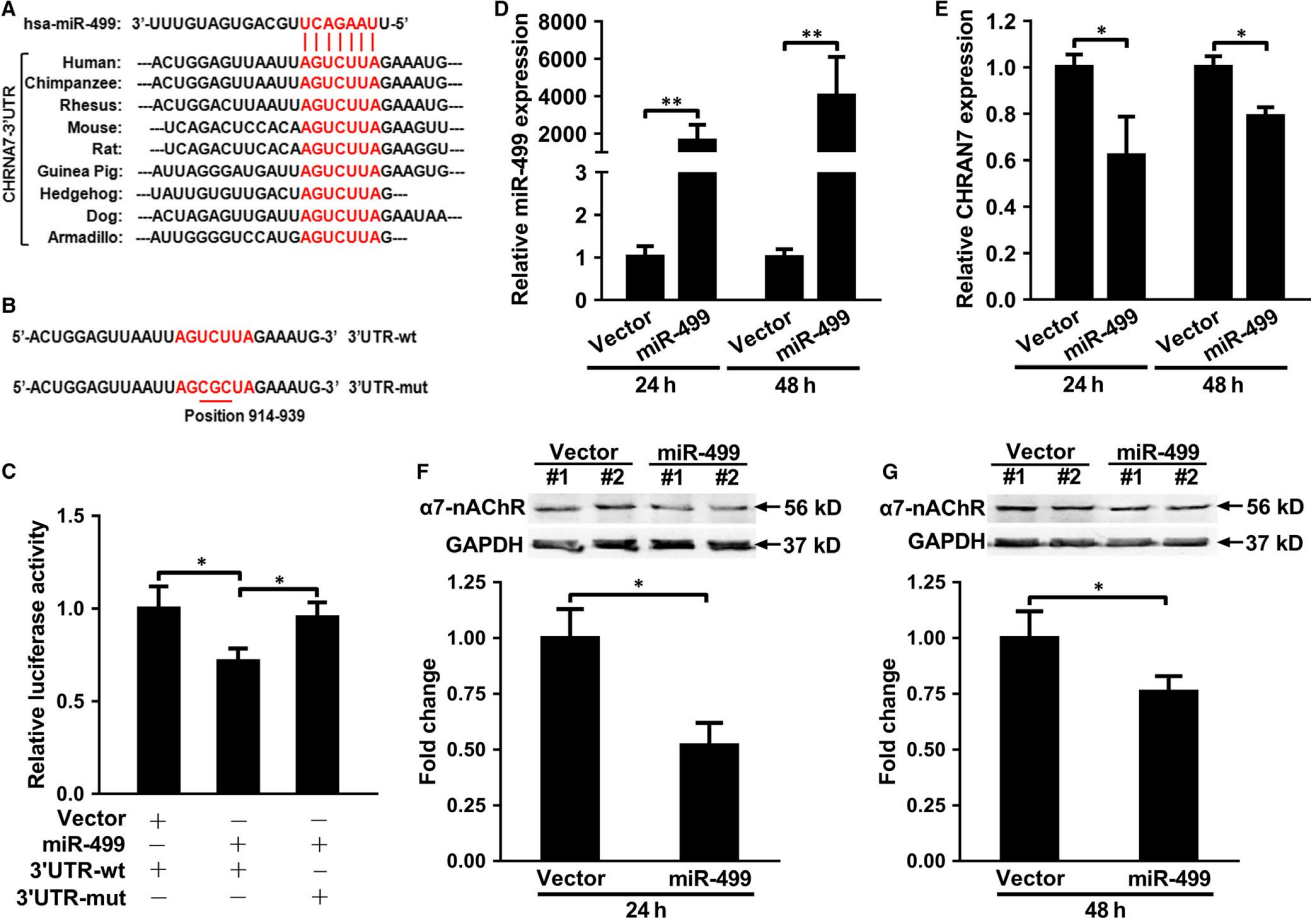
CHRNA7 is a target of hsa‐miR‐499. A, The ‘seed sequence’ of hsa‐miR‐499 is complementary to the target sequence of 3′UTR of CHRNA7 that is conserved amongst species. B, The ‘target sequence’ of human CHRNA7 was mutated from AGUCUUA (wt, top) to AGCGCUA (mut, bottom) with mutated bases underlined. C, miR‐499 significantly decreased the luciferase activity of 3′UTR‐wt compared to Vector, which was abolished by the mutation of the target sequence (3′UTR‐mut). D, miR‐499 expression was significantly higher in hsa‐miR‐499 than vector transfected A549 cells at 24 and 48 h respectively (n = 3 and *P* < 0.01 for both). E, miR‐499 significantly decreased CHRNA7 expression compared to vector transfection at 24 and 48 h respectively (n = 3 and *P* < 0.05 for both). F, Representative image of Western Blot showing the effect of transient transfection of Vector versus miR‐499 on the expression of α7‐nAchR expression (top panel) and that of GAPDH (bottom panel), and quantification that miR‐499 significantly decreased the expression of α7‐nAchR than that of Vector (bottom panel) at 24 h (n = 2 and *P* < 0.05). G, Representative image of Western Blot showing the effect of transient transfection of Vector versus miR‐499 on the expression of α7‐nAchR expression (top panel) and that of GAPDH (bottom panel), and quantification that miR‐499 significantly decreased the expression of α7‐nAchR than that of Vector (bottom panel) at 48 h (n = 2 and *P* < 0.05). Luciferase activity was normalized to that of Renilla activity. All experiments were in triplicate and repeated at least three times. The expression of miR‐499 and CHRNA7 was normalized to that of U6 RNA and GAPDH respectively in qPCR and the expression of α7‐nAchR was normalized to that of GAPDH in Western blot. Data are presented as ‘Mean ± SD’. The comparison between two groups was performed using paired or non‐paired Student's *t* test, with * denoting *P* < 0.05 and ** *P* < 0.01

**Figure 3:**
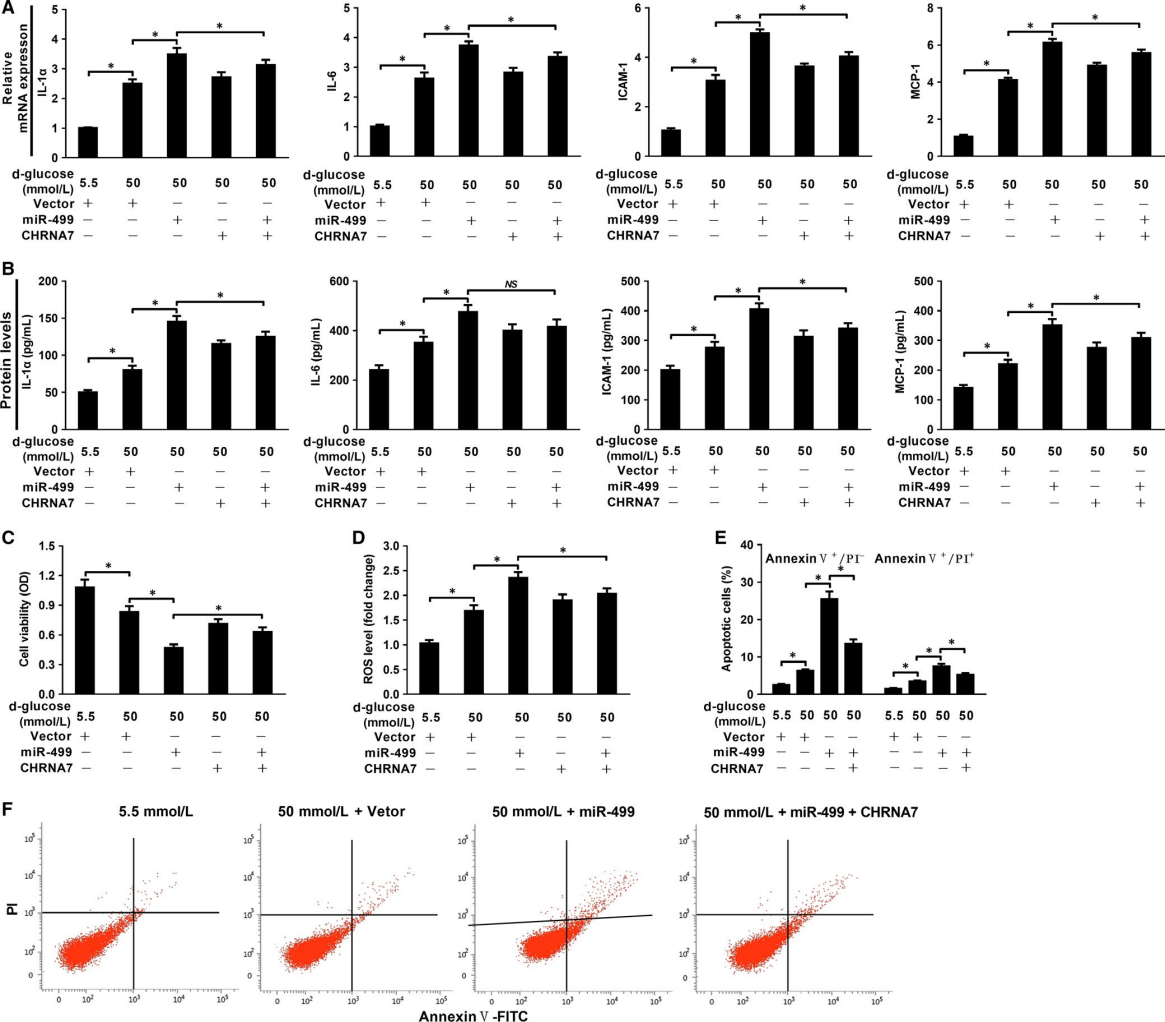
miR‐499 aggravates high glucose‐induced RPMECs injury, which could be partially ameliorated by CHRNA7 overexpression. miR‐499 further increased the high glucose‐induced up‐regulation of ICAM‐1, MCP‐1, IL1α and IL‐6 at mRNA level (n = 3 and *P* < 0.05) (A) and protein level (n = 3 and *P* < 0.05) (B), which could be ameliorated by forced expression of CHRNA7. miR‐499 overexpression further worsened the cell survival by high glucose, which could also be partially reversed by CHRNA7 overexpression as evaluated by CCK‐8 assay (n = 3 and *P* < 0.05) (C) and ROS analysis (n = 3 and *P* < 0.05) (D). FACS plots (E) and quantification (F) showing that miR‐499 further increased the percentage of apoptotic cells compared with that of control, which was also partially reversed by forced expression of CHRNA7 (n = 3 and *P* < 0.05). Experiments were performed three times in triplicate and data are presented as ‘Mean ± SD’. The comparison between two groups was performed using paired or non‐paired Student's *t* test, with *NS* denoting not significant and * *P* < 0.05

The ‘seed sequence’ of miR‐499 is complementary to the 3′UTR of CHRNA7 from 928 to 935 downstream of the stop codon TAA, which is conserved amongst species listed (Figure 2A). The 3′UTR ranging from 760 to 1016 was amplified by PCR and cloned into psiCHECK2, and the core of the target sequence was mutated from AGUCUUA to AGCGCUA (Figure 2B). Dual luciferase assay showed that miR‐499 decreased the luciferase activity of the 3′UTR‐wt to 75% of pcDNA3.1 (Vector) transfection, and mutation of the target sequence (Figures 2B, 3′UTR‐mut) abrogated miR‐499‐mediated down‐regulation of luciferase activity (Figure 2C). To prove that miR‐499 regulates endogenous α7‐nAChR expression, miR‐499 was transiently transfected into A549 cells, a human adenocarcinomic alveolar basal epithelial cell line that expresses high level of α7‐nAChR.^26^ Over‐expression of miR‐499 (Figure 2D) significantly decreased the expression of both CHRNA7 (Figure 2E) and α7‐nAChR (Figure 2F,G) compared to Vector (Figure 2E,G) at both 24 and 48 hours. Similar results were obtained on PC12 cells, a cell line derived from pheochromocytoma of rat adrenal medulla that also expresses high level of α7‐nAChR (Figure S1A‐1C). Taken together, our observations indicate that CHRNA7 is a target of miR‐499.

### 3.4 miR‐499 aggravates high glucose‐induced endothelial injury by targeting CHRNA7

ECs express α7‐nAChR that helps to maintain the homoeostasis of endothelium.^27^ To simulate endothelial cell injury in vitro, rat pulmonary microvascular endothelial cells (RPMECs) were treated with various concentrations of D‐glucose as indicated, it dose‐dependently up‐regulates the expression of IL‐1α, IL‐6, IL‐8, TNFα, ICAM‐1 and MCP‐1 (Figure S2A), induces decreased cell viability (Figure S2B), and increases ROS production (Figure S2C). Since 50 mM D‐glucose up‐regulated all pro‐inflammatory cytokines mentioned above, cell apoptosis was analysed by fluorescence‐activated cell sorting (FACS). As shown in Figure S2D and E, 50 mM D‐glucose resulted in significantly increased apoptotic cells compared to 5.5 mM D‐glucose. To explore the functional roles of miR‐499 and α7‐nAchR in endothelial injury, RPMECs were transiently transfected with Vector, miR‐499, or CHRNA7 in the presence of 50 mM D‐glucose as indicated below X‐axis, and forced expression of miR‐499 (Figure S2F) and CHRNA7 (Figure S2G) significantly increased, the levels of IL‐1α, IL‐6, ICAM‐1 and MCP‐1 at both mRNA (Figure 3A) and protein levels (Figure 3B) compared to that of vector transfection. Furthermore, forced expression of miR‐499 significantly decreased cell viability (Figure 3C), increased ROS (Figure 3D) production, and worsened cell apoptosis (Figure 3E,F) compared to vector control, which was partially abrogated by forced expression of CHRNA7. These results indicate that miR‐499 aggravates high glucose‐induced endothelial injury by targeting α7‐nAchR.

### 3.5 miR‐499 released from injured NRCMs internalizes into RPMECs and aggravates glucose‐induced endothelial cell injury

**Figure 4:**
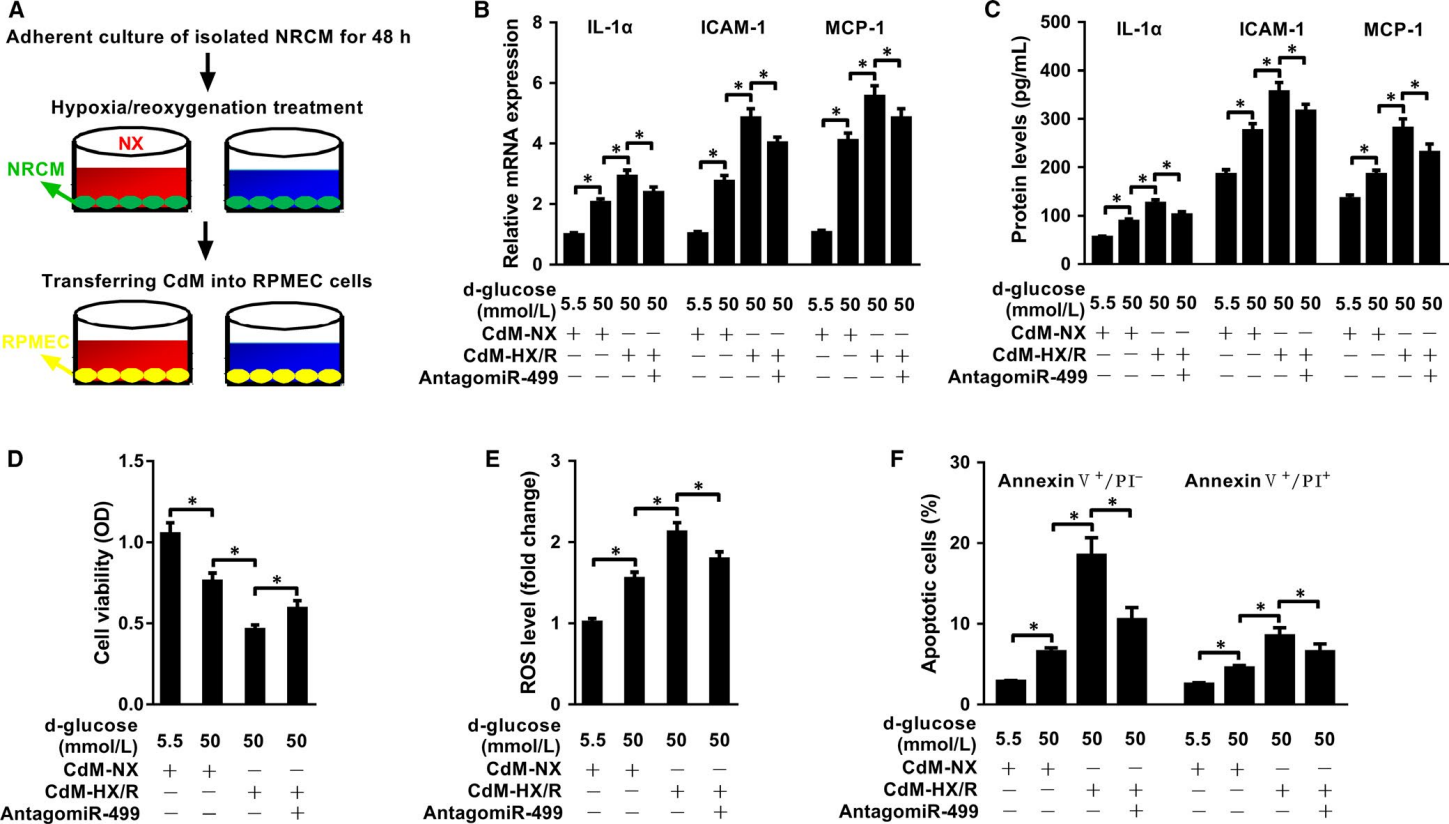
miR‐499 from injured NRCMs internalizes into RPMECs and worsens high glucose‐induced injury. A, Cartoon showing indirect co‐culture model of NRCMs and RPMECs using CdM. The CdM from NRCMs subjected to HX/R treatment significantly increased the expression levels of IL‐1α, ICAM‐1 and MCP‐1 both at mRNA and protein levels as evaluated by qRT‐PCR. B, (n = 3 and *P* < 0.05 for all) and ELISA. C, (n = 3 and *P* < 0.05 for all), which could be significantly blocked by AntagomiR‐499. D, CCK‐8 analysis showed that the CdM from NRCMs subjected to HX/R further decreased the cell viability induced by high glucose, which could be ameliorated by down‐regulation of the endogenous miR‐499 in NRCMs (n = 3 and *P* < 0.05 for all). E, ROS assay showed that the CdM from NRCMs subjected to HX/R further increased the ROS levels of RPMECs caused by high glucose, which could be partially inhibited by antagomiR‐499 (n = 3 and *P* < 0.05 for all). Experiments were performed three times in triplicate, and data are presented as ‘Mean ± SD’. The comparison between two groups was performed using paired or non‐paired Student's *t* test, with * denoting *P* < 0.05

To mimic the cross‐talk between CMs and endothelial cells during AMI in vitro, NRMCs were allowed to attach for 48 hours, refreshed with serum‐free medium and kept at 0.1% oxygen for 4 hours and the cells were then switched to the complete medium under normoxia for 2 hours for the experimental group, while the control cells were kept in the complete medium under normoxia. The CdM was then added to the pre‐attached RPMECs in the presence of 50 mM D‐glucose, and the cells were continued to incubate for 24 hours (Figure 4A). HX/R treatment significantly decreased the ratio of live cells (Figure S3A), inhibited cell viability (Figure S3B), increased LDH release (Figure S3C) and promoted cell apoptosis (Figure S3D, E), compared to the NRCMs treated with NX. When the CdM was analysed, it contained significantly higher levels of miR‐499 in NRCMs subjected to HX/R than to NX, which was significantly neutralized by antagomiR‐499 (Figure S3F). When RPMECs were incubated with the CdM, it significantly increased the level of miR‐499 of RPMECs by the CdM from HX/R than from NX, which was significantly inhibited by addition of antagomiR‐499 (Figure S3G). However, when RPMECs were incubated with the CdM from RPMECs subjected to HX/R, it did not have an effect on the level of miR‐499 compared cells treated NX in the presence of either 5.5 or 50 mM D‐glucose (Figure S3H), arguing against the increased miR‐499 in RPMECs was from up‐regulated endogenous miR‐499. Consistent with the increased miR‐499 in CdM from HX/R, when incubated with RPMECs, it significantly increased the levels of IL‐1α, ICAM‐1 and MCP‐1 at both mRNA (Figure 4B) and protein (Figure 4C) levels compared to the CdM from NX, which were significantly inhibited by the addition of antago miR‐499. When cell injury was analysed, the CdM from HX/R significantly decreased cell viability (Figure 4C), increased production of ROS (Figure 4D), and increased the percentage of early (Annexin V^+^/PI^−^) and late stages (Annexin V^+^/PI^+^) of apoptotic cells (Figure 4E) compared to the CdM from NX in the presence of 50 mM D‐glucose, which were significantly reversed by addition of antagomiR‐499.

### 3.6 The miR‐499 free from exosome fraction internalizes into endothelial cells

**Figure 5:**
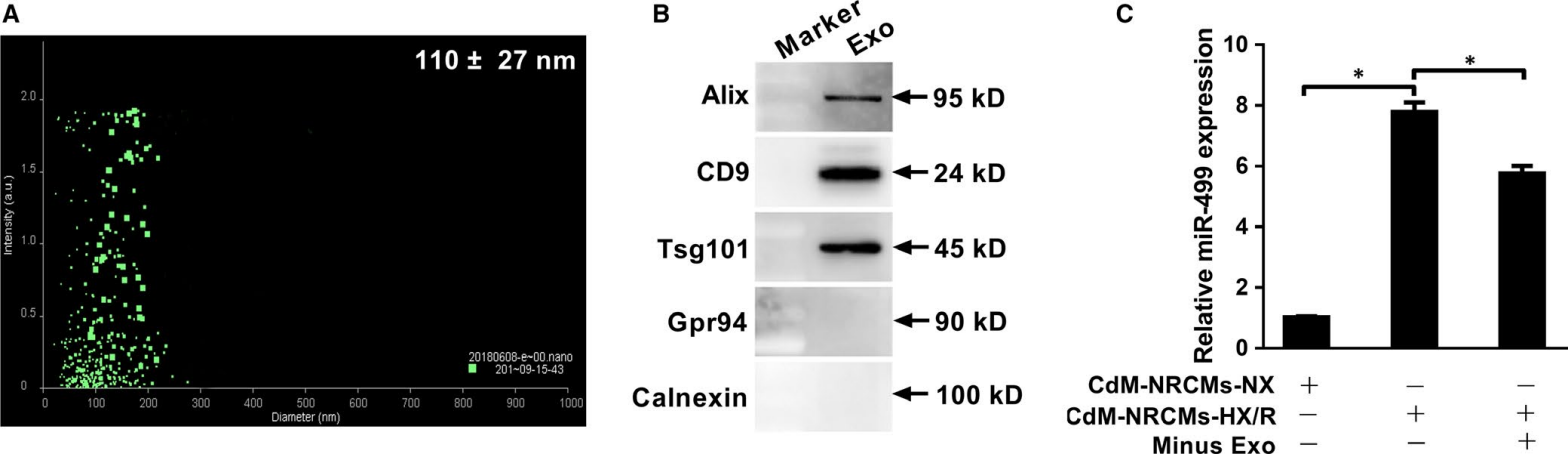
Free miR‐499, not exosome‐associated miR‐499, released from injured NRCMs accounts for increased level of miR‐499 in RPMEC cells. A, Exosomes of NRCMs are 110 ± 27 nm in diameter by NanoSight NS300. B, Exosomes are positive for Alix, CD9 and Tsg101 and negative for Grp94 and calnexin. C, The CdM from NRCMs subjected to HX/R significantly increased miR‐499 in RPMEC cells compared to the that from NX control, and depletion of exosomes from CdM‐HX/R decreased miR‐499 expression by 25% (n = 3 and *P* < 0.05 for all). The expression of miR‐499 was normalized to that of U6 RNA. Experiments were performed three times in triplicate. Data are presented as ‘Mean ± SD’. Two groups comparison was performed using paired or non‐paired Student's *t* test, with * denoting *P* < 0.05

Exosomes carrying miRNAs have been shown to mediate cell‐cell communication. We tested whether miR‐499 released from injured CMs was carried by exosome. Exosomes and the corresponding exosome‐free CdM were prepared from NRCMs subjected to HX/R, which were incubated with RPMECs respectively for 24 hours. Exosomes were 110 ± 27 nm in diameter by NanoSight 3000 (Figure 5A), positive for exosome markers Alix, CD9 and Tsg101, and negative for endoplasmic reticulum markers Grp94 and Calnexin (Figure 5B). When CdM and exosome‐depleted CdM were incubated with RPMECs, although both fractions significantly increased the level of miR‐499 in RPMECs compared to the CdM from NX, the CdM induced a much higher increase of miR‐499 than that of exosome‐depleted CdM (Figure 5C), suggesting the miR‐499 in the CdM‐HX/R was mainly carried in exosome‐free form.

### 3.7 Perfusate from I/R impairs endothelium‐dependent relaxation

**Figure 6:**
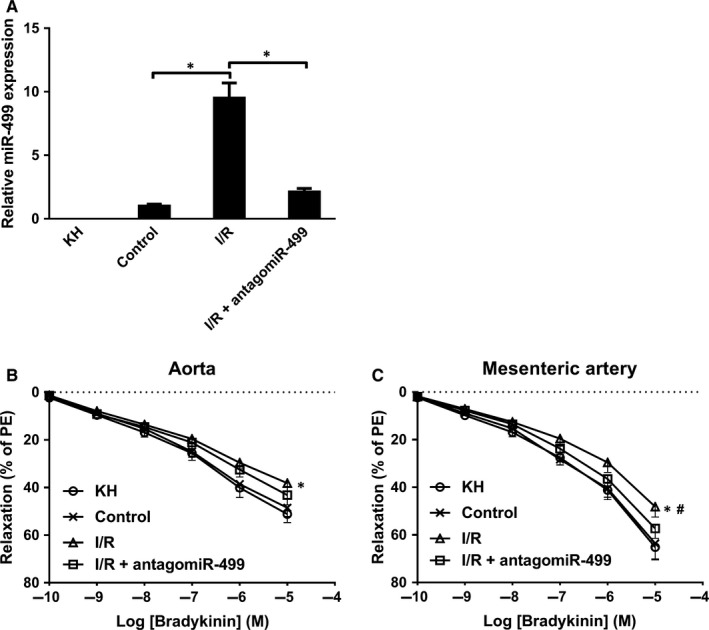
Perfusate from I/R impairs endothelium‐dependent relaxation. A, The amount of miR‐499 was significantly higher in I/R compared to control perfusate, non‐detectable in KH, and significantly decreased by addition of antagomiR‐499 (n = 3 and *P* < 0.05 for all). B, The I/R perfusate (△) decreased ~21% and ~26% of EDR at 10^−5^ and 10^−6 ^M of Bradykinin, respectively, relative to control (×) and KH buffer (○) in aorta (n = 3 and *P* < 0.05), and addition of antagomiR‐499 (□) tends to restore EDR (n = 3 and *P* > 0.05). C, The I/R (△) decreased ~25%, ~27% and ~29% of EDR at 10^−5^ 10^−6^, and 10^−7^ M of Bradykinin, respectively, relative to control (×) and KH buffer (○) in mesenteric artery (n = 3 and *P* < 0.05), and antagomiR‐499 (□) abrogated the miR‐499‐mdiated decrease of EDR (^#^compared to I/R, n = 3 and *P* < 0.05). Experiments were performed three times in triplicate. Data are presented as ‘Mean ± SEM’. Dose‐response curves were analysed with 2‐way analysis of variance, with * denoting *P* < 0.05 and ^#^ *P* < 0.05.

To give more insight into the functional role of myocardium‐derived miR‐499 in endothelial function, adult rat heart was perfused in a Langendorff apparatus, and the perfusate before (control) and after I/R were collected and then incubated with arterial rings for endothelium‐dependent relaxation (EDR). The level of miR‐499 was significantly higher in I/R perfusate than control and KH buffer (KH), and was significantly neutralized by antagomiR‐499 (Figure 6A). When incubated with arteries, the I/R perfusate significantly impaired the Bradykinin‐mediated EDR in aorta (Figure 6B) and mesenteric artery (Figure 6C), which was significantly abrogated by antagomiR‐499 in mesenteric artery (Figure 6C), but not in aorta (Figure 6B). These results suggest that miR‐499 contributed to the injury of endothelium during AMI.

### 3.8 Levels of plasma miR‐499 are directly correlated with endothelium injury

**Figure 7:**
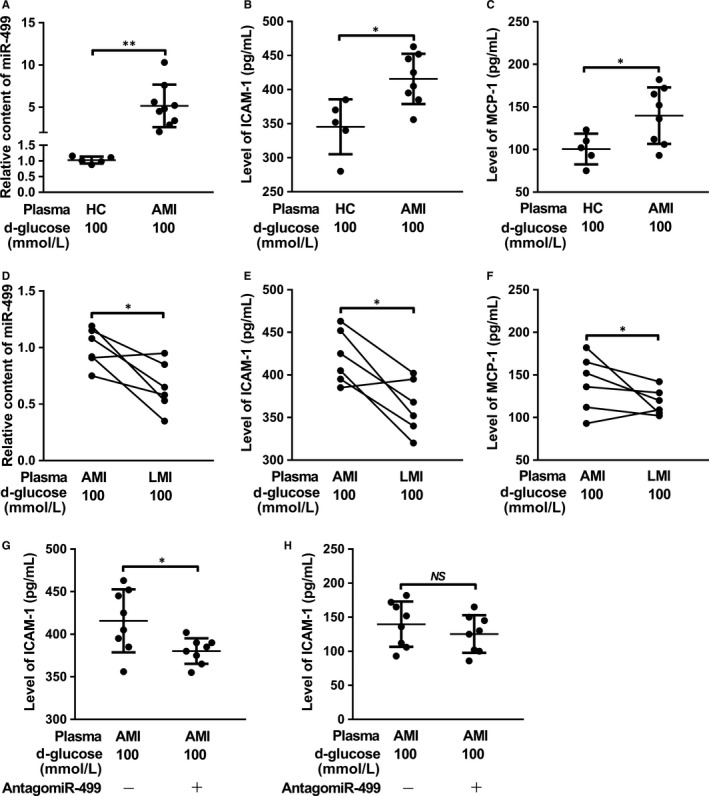
Plasma of patients with AMI aggravates high glucose‐induced endothelial damage. After incubation with plasma, the levels of miR‐499 in HUVECs were significantly higher in AMI than HC (n = 5 in HC, n = 8 in AMI and *P* < 0.01) (A) and LMI (n = 6 in each and *P* < 0.05) (D) respectively. When incubated with RPMECs in the presence of 100 mM glucose, plasma from AMI patients further increased the levels of ICAM‐1 (B, E) and MCP‐1 (C, F) compared to that of HC and LMI (n = 5 in HC, n = 8 in AMI and *P* < 0.01 for B and C) (n = 6 in each and *P* < 0.05 for E and F), respectively. AntagomiR‐499 could partially abrogate the plasma miR‐499‐mediated increased ICAM‐1 (n = 8 in each and *P* < 0.05) (G) and MCP‐1 (n = 8 in each and *P* > 0.05 (H). The expression of miR‐499 was normalized to that of U6 RNA. Data are presented as ‘Mean ± SD’. Mann‐Whitney *U* test (A‐C, G, H) and Wilcoxon test (D‐F) were used for statistical analysis, with * denoting *P* < 0.05, ** *P* < 0.01 and *NS* denoting not significant.

We have showed that plasma miR‐499 was directly correlated with endothelium injury in post‐AMI patients (Figure 1). To show that miR‐499 released from injured CMs may cause endothelial injury, plasma from patients with AMI, LMI and HC were incubated with HUVECs, and levels of the pro‐inflammatory cytokines in the CdM were analysed. As shown in Figure 7, plasma from AMI, when incubated with HUVECs, significantly increased the levels of miR‐499 (7A, 7D), secreted ICAM‐1 (7B, 7E), and secreted MCP‐1 (7C, 7F) compared to the plasma from HC and LMI respectively in the presence of 100 mM D‐glucose, and antagomiR‐499 significantly decreased the levels of the secreted ICAM‐1 (Figure 7G), but not MCP‐1 (Figure 7H).

To further validate that plasma miR‐499 is correlated with endothelial injury, HUVECs were incubated with plasma from another cohort of T2DM patients without or with AMI (T2DM/AMI). Plasma contained significantly higher levels of miR‐499 in patients with T2DM/AMI than with T2DM (Figure S4A). When endothelial cell injury was analysed, plasma levels of TM were significantly higher in T2DM/AMI than T2DM (Figure S4B), and plasma levels of TM were directly correlated with the levels of miR‐499 (Figure S4C). When serum was incubated with HUVECs, the plasma from T2DM/AMI significantly increased the levels of miR‐499 (Figure S4D), levels of ICAM‐1(Figure S4E) and levels of MCP‐1 (Figure S4F) in HUVECs compared to that from T2DM.

## 4 DISCUSSION

In this report, we showed that plasma miR‐499, a marker for cardiomyocyte injury, is significantly increased in patients immediately after AMI, which is directly correlated with plasma TM, a marker for endothelial cell injury. Plasma of AMI patients, when incubated with rat RPMECs, significantly increased the levels of endothelial injury markers, which could be abrogated by antagomiR‐499. In vitro, NRCMs subjected to HX/R release exosome‐free miR‐499 that can be internalized into RPMECs, leading to their injury. In silico analysis demonstrated that CHRNA7 is a target of miR‐499, which was validated in cell lines expressing high levels of endogenous α7‐nAchR. In D‐glucose‐induced RPMEC injury model, miR‐499 aggravated the injury that could be ameliorated by antagomiR‐499 and α7‐nAchR. Moreover, the perfusate from Langendorff perfused heart during I/R contained higher amount of miR‐499 that significantly impaired the Bradykinin‐mediated EDR in rat aorta and mesenteric artery, which could be alleviated by antagomiR‐499. Finally, the correlation between miR‐499 and EC injury was validated in another cohort of patients with AMI. These discoveries indicate that miR‐499 released from I/R injured CMs contributes to the dysfunction of endothelium by targeting α7‐nAchR, exaggerating vascular inflammation after AMI.

Endothelium dysfunction upon reperfusion is usually resulted from injuries of both ECs and CMs. The former comes from ischaemia‐induced activation of innate immune system, the imbalance of excessive production of ROS versus the inhibition of nitric oxide synthase, the increased vasoconstrictor versus decreased vasodilator production, vascular remodelling, increased cellular apoptosis and the continued increased expression of adhesion molecules.^28^ Reperfusion of CMs after AMI is also associated with the increased production of ROS, leading to oxidative stress that causes cellular injury including CMs and ECs.^4^ In addition, the injured CMs also release DAMPs and other signal molecules such as miRNAs that can diffuse to ECs causing endothelial injury.^7^, ^8^ These CM‐derived miRNAs can be either naked or associated with extracellular vesicles. For example, Li et al demonstrated that miR‐939 derived from CMs during AMI promoted angiogenesis through targeting inducible nitric oxide synthase (iNOS) gene, and this miR‐939 was associated with exosomes.^29^ In contrast, Zhang et al showed miR‐499 released from infarcted myocardium was free from exosomes, which initiated EC inflammation through targeting PDCD4.^12^, ^14^ In agreement with the later report, the increased plasma miR‐499 immediately after AMI (Figure 1A,C) exists in the exosome‐free form since exosome depleted CdM, when incubated with RPMECs, significantly increased the miR‐499 level compared to exosome fraction in our NRCM‐HX/R injury model (Figure 5C,D).

CAIP is a newly discovered neuroimmune signalling pathway that suppresses peripheral inflammation through vagus nerve circuit.^15^ The terminal regulator of the CAIP is α7‐nAchR expressed on the surface of macrophages, which, upon activation, inhibits the release of pro‐inflammatory cytokines through spleen‐dependent and/or spleen‐independent pathways, and thus dampening the original peripheral inflammation and ameliorating organ ischaemia/reperfusion injury.^17^, ^30^, ^31^, ^32^ α7‐nAchR is also expressed on the surface of ECs, which has been shown to help maintain the integrity of endothelium and modulate the recruitment of immune cells during inflammation.^33^, ^34^, ^35^, ^36^ For example, TNF‐α induced endothelial barrier dysfunction and increased recruitment of leukocytes could be prevented by acetylcholine agonists in a dose‐dependent manner, and this protective effect could be abolished by α7‐nAchR‐specific antagonist, α‐bungarotoxin.^37^ Oxidized LDL‐ and pathogen‐induced expression of adhesion molecule on ECs could be inhibited by nicotine and other cholinergic mimetics via up‐regulation of α7‐nAchR.^27^, ^37^, ^38^, ^39^ Using in silico analysis, luciferase assay and gene expression analysis, we showed that α7‐nAchR is a direct target of miR‐499 (Figures 2, 3, and Figure S1). Consistently, in the glucose‐induced HUVEC injury model, miR‐499 targeting CHRNA7 (Figure 2 and Figure S1) significantly increased the glucose‐induced expression of ICAM‐1, MCP‐1 and IL‐6 at mRNA (Figure 3A‐C) and ICAM‐1 and MCP‐1 at protein levels (Figure 3D,E), which could be ameliorated by forced expression of CHRNA7 and by antagomiR‐499. Of functional importance, the perfusate from I/R contained higher amount of miR‐499 (Figure 6A) and significantly impaired the Bradykinin‐mediated EDR that could be restored by antagomiT‐499 (Figure 6B,C), suggesting that elevated miR‐499 released from I/R mediates the endothelial injury, despite we could not exclude other mediators released during reperfusion. Furthermore, the plasma of patients with AMI that contained higher amount of miR‐499 significantly increased endothelial levels of miR‐499 and pro‐inflammatory cytokines, aggravating the high glucose‐induced endothelial injury.

In summary, the study presented here uncovered a molecular mechanism that contributes to the surged inflammation of endothelium immediately after AMI. Our working hypothesis shown in graphic abstract is that miR‐499 released from injured CMs is internalized into ECs, where it down‐regulates α7‐nAchR, dampening α7‐nAchR‐mediated CAIP through activation of ECs and thus aggravating endothelium injury.

## 5 CONCLUSIONS

miR‐499 released during AMI aggravates endothelial cell injury through targeting α7‐nAchR, suggesting that targeting miR‐499/α7‐nAchR pathway may alleviate the surged vascular inflammation immediately after AMI.

## CONFLICT OF INTEREST

The authors have no conflict of interest to disclose.

## AUTHOR'S CONTRIBUTION

The research was conceived by XD, RZ and CD, experiments were performed and data were analysed by WH, XF, FL, LL, HY, YJ, HX and ZZ, and manuscript was drafted by X. D. and edited by C. D. and R. Z.

## Data Availability

All data are available upon request.
