# Supplementary figures and images for: miR‐499 released during myocardial infarction causes endothelial injury by targeting α7‐nAchR
Source: J Cell Mol Med. 2019 Jul 3;23(9):6085–97. doi: 10.1111/jcmm.14474 (PMC6714230; doi:10.1111/jcmm.14474)

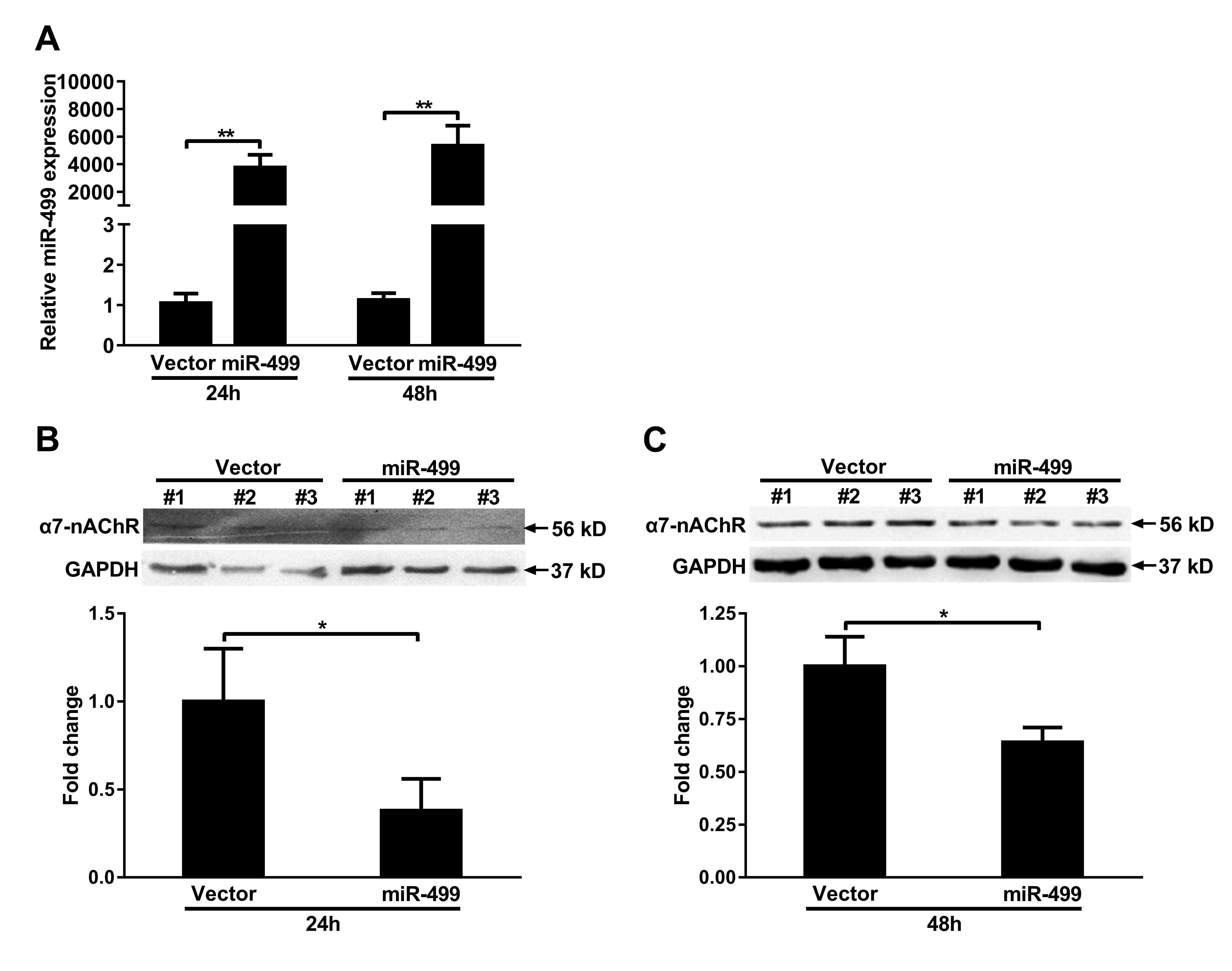

Supplement: Supplementary file 1 [file JCMM-23-6085-s001.tif]

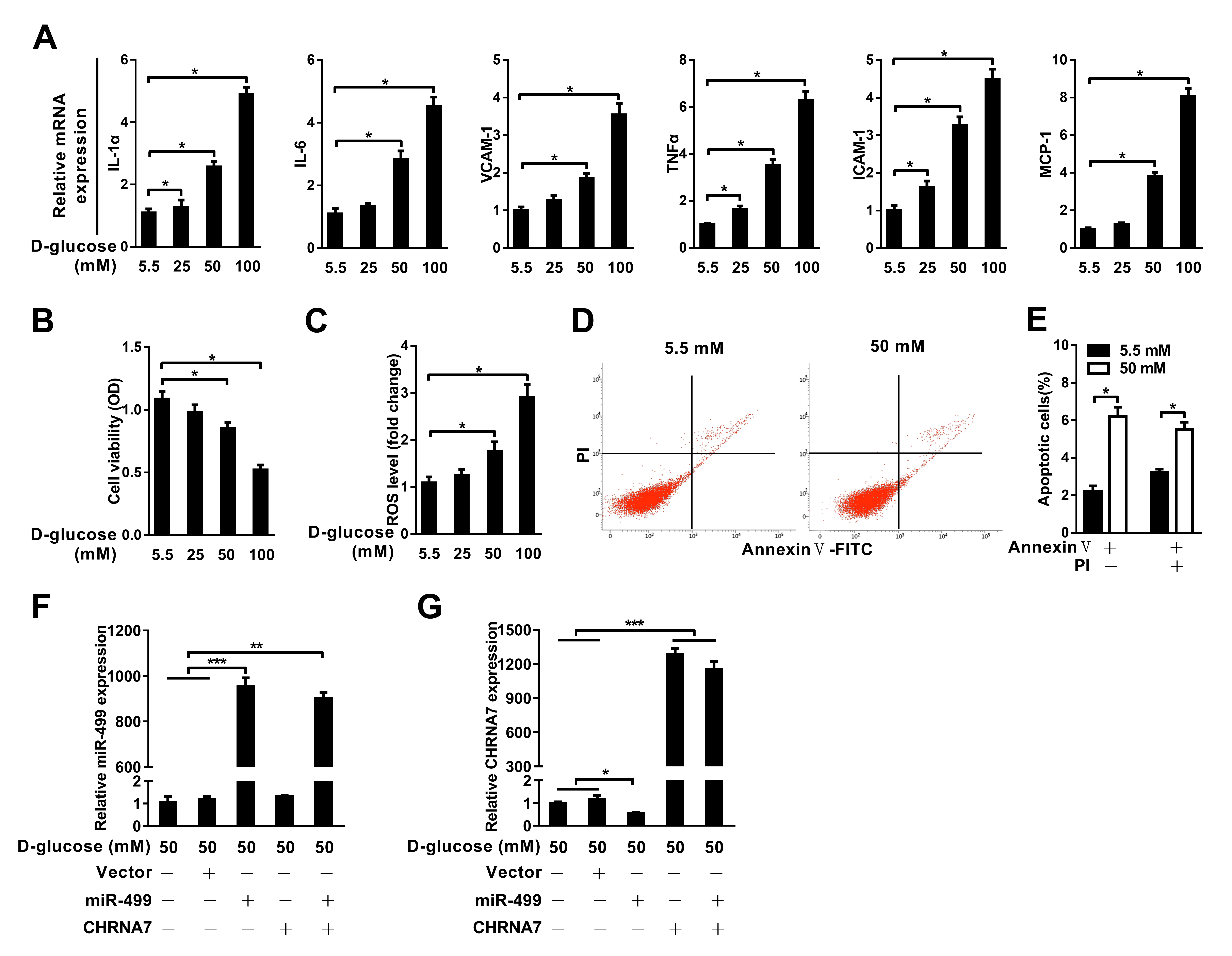

Supplement: Supplementary file 2 [file JCMM-23-6085-s002.tif]

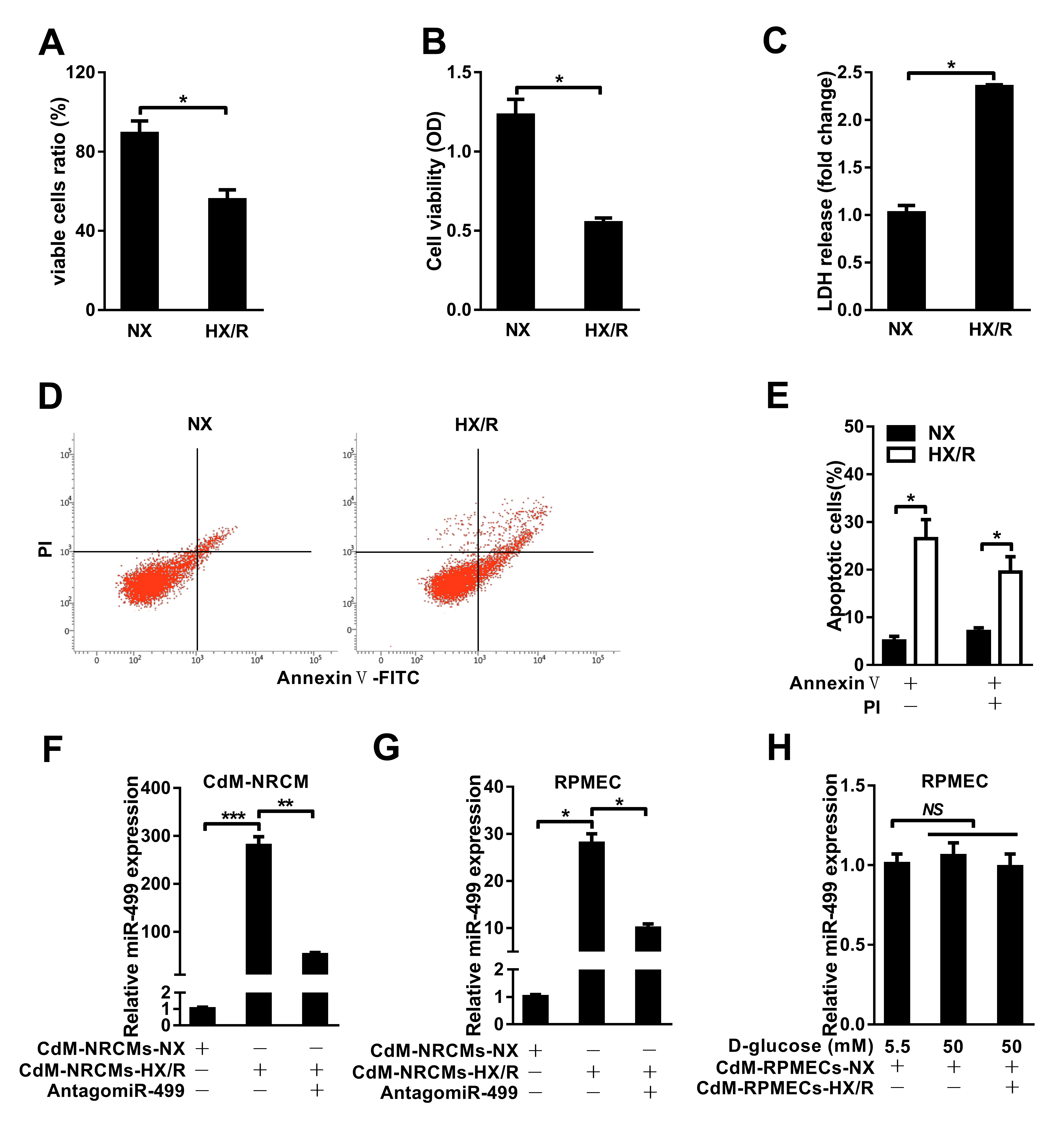

Supplement: Supplementary file 3 [file JCMM-23-6085-s003.tif]

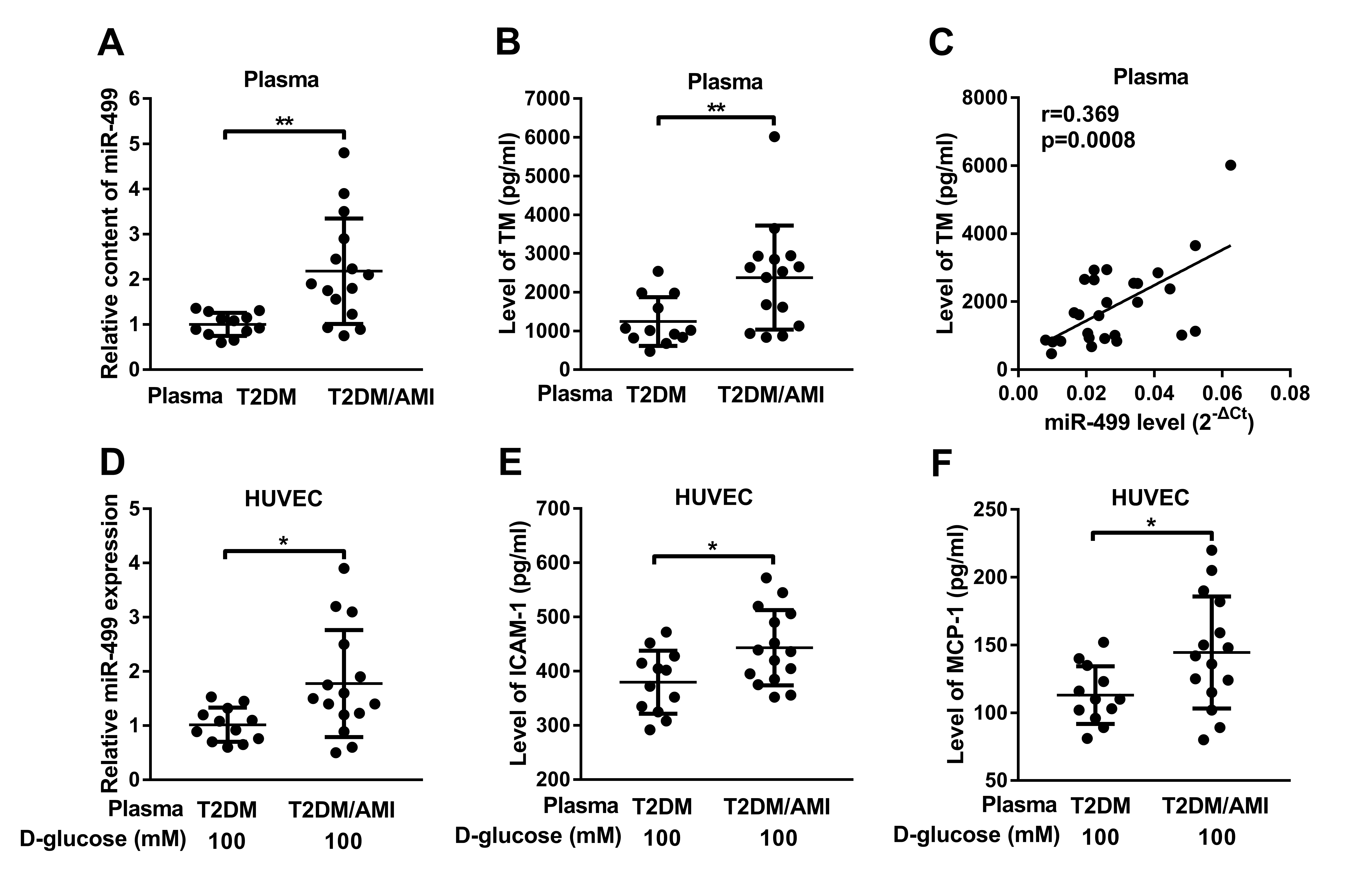

Supplement: Supplementary file 4 [file JCMM-23-6085-s004.tif]
