# Supplementary material for: miR‐499 released during myocardial infarction causes endothelial injury by targeting α7‐nAchR
Source: J Cell Mol Med. 2019 Jul 3;23(9):6085–97. doi: 10.1111/jcmm.14474 (PMC6714230; doi:10.1111/jcmm.14474)
Supplement: Supplementary file 5 [file JCMM-23-6085-s005.docx]

Table1. Primer sets for qRT-PCR analysis.

| Gene | Direction | Sequence (5’-3’) | Product size (bp) |
| --- | --- | --- | --- |
| rIL-1α^4^ | Forward | CCTCGTCCTAAGTCACTCGC | 102 |
|  | Reverse | GGCTGGTTCCACTAGGCTTT |  |
| rIL-6 | Forward | AGCGATGATGCACTGTCAGA | 127 |
|  | Reverse | GGAACTCCAGAAGACCAGAGC |  |
| rVCAM-1 | Forward | GGAAATGCCACCCTCACCTT | 156 |
|  | Reverse | CACCTGAGATCCAGGGGAGA |  |
| rTNFα | Forward | CATCCGTTCTCTACCCAGCC | 146 |
|  | Reverse | AATTCTGAGCCCGGAGTTGG |  |
| rICAM-1 | Forward | GCCTGGGGTTGGAGACTAAC | 91 |
|  | Reverse | CTGTCTTCCCCAATGTCGCT |  |
| rMCP-1 | Forward | CAGGTCTCTGTCACGCTTCT | 87 |
|  | Reverse | GGCATTAACTGCATCTGGCTG |  |
| hCHRNA7^5^ | Forward | CTCCTGCACGGTAAAGCCA | 111 |
|  | Reverse | GCCTTGCAGGGACACTGG |  |
| Hsa-miR-499/  Rno-miR-499 | RT^1^ | GTCGTATCCAGTGCAGGGTCCGAGGTATTCGCACTGGATACGACAAACATC | 61 |
|  | Forward | GCGAGCTTAAGACTTGCAGT |  |
|  | Reverse | GTGCAGGGTCCGAGGT |  |
| Hsa-miR-103a | RT | GTCGTATCCAGTGCAGGGTCCGAGGTATTCGCACTGGATACGACTCATAG | 61 |
|  | Forward | GCACAGCAGCATTGTACAGGG |  |
|  | Reverse | GTGCAGGGTCCGAGGT |  |
| hU6 | RT | GTCGTATCCAGTGCAGGGTCCGAGGTATTCGCACTGGATACGACAAAATATG | 60 |
|  | Forward | GCGCGTCGTGAAGCGTTC |  |
|  | Reverse | GTGCAGGGTCCGAGGT |  |
| hCHRNA7-  3’UTR-wt | Forward | TCATCTCGAGGTGCTGCTCTCAGACACT | 256 |
|  | Reverse | TACTGCGGCCGCAGGTATCCAGAGAAGGGATG |  |
| hCHRNA7-  3’UTR-mut | Forward^2^ | TCATCTCGAGGTGCTGCTCTCAGACACT | 256 |
|  | Reverse^2^ | TACTGCGGCCGCAGGTATCCAGAGAAGGGATG |  |
|  | Forward^3^ | ATGCACTGGAGTTAATTAGCGCTAGAAATGTGTGCATCCA |  |
|  | Reverse^3^ | TGGATGCACACATTTCTAGCGCTAATTAACTCCAGTGCAT |  |

^1^ RT, Reverse transcription primer for miRNA. ^2^ Outer primer for mutation of CHRNA7-3’UTR-wt. ^3^ inner primer for mutation of CHRNA7-3’UTR-wt. ^4^ rIL-1α, rat IL-1α. ^5^ hCHRNA7, human CHRNA7.
