# Supplementary material for: miR‐499 released during myocardial infarction causes endothelial injury by targeting α7‐nAchR
Source: J Cell Mol Med. 2019 Jul 3;23(9):6085–97. doi: 10.1111/jcmm.14474 (PMC6714230; doi:10.1111/jcmm.14474)
Supplement: Supplementary file 6 [file JCMM-23-6085-s006.docx]

| Variables | HC (n=5) | AMI (n=9) |
| --- | --- | --- |
| Age (years) | 57 ± 7 | 63 ± 6 |
| No. of males/female | 2/3 | 6/3 |
| BMI (kg/m^2^) | 24.1 ± 1.9 | 28.3 ± 3.4* |
| Systolic BP (mmHg) | 126 ± 22 | 132 ± 25 |
| Diastolic BP (mmHg) | 84 ± 21 | 87 ± 17 |
| No. of smokers | 0 | 4 |
| Hemoglobin (g/L) | 147 ± 15 | 138 ± 22 |
| Creatinine (μmol/L) | 64 ± 23 | 74 ± 30 |
| Triglycerides (mmol/L) | 1.05 ± 0.4 | 1.3 ± 0.5 |
| Total cholesterol (mmol/L) | 3.5 ± 0.6 | 6.5 ± 0.7** |
| HDL (mmol/L) | 1.7 ± 0.4 | 1.0 ± 0.3* |
| LDL (mmol/L) | 3.4 ± 0.7 | 4.9 ± 0.8* |
| hs-TNT (ng/ml) | 3.3 ± 0.7 | 63.2 ± 10.4*** |
| MYO (μg/ml ) | 26 ± 12 | 2178 ± 265*** |
| CK-MB (μg/ml ) | 2.2 ± 0.7 | 236 ± 35*** |
| Aspirin | 0 | 9 |
| Lipid lowering medication | 0 | 9 |
| PCI | 0 | 9 |

Table 2. Basic characteristics of patients with or without AMI.

HC, healthy control; AMI, acute myocardial infarction; BMI, body mass index; BP, blood pressure; HDL, high-density lipoprotein; LDL, low-density lipoprotein; hs-cTNT, highly sensitive cardiac troponin T; MYO, Myoglobin; CK-MB, creatine kinase-muscle/brain; PCI, Percutaneous coronary intervention. Data are expressed as mean ± SD; * *p* < 0.05, ** *p* < 0.01, *** *p* < 0.001.
