# Supplementary material for: miR‐499 released during myocardial infarction causes endothelial injury by targeting α7‐nAchR
Source: J Cell Mol Med. 2019 Jul 3;23(9):6085–97. doi: 10.1111/jcmm.14474 (PMC6714230; doi:10.1111/jcmm.14474)
Supplement: Supplementary file 7 [file JCMM-23-6085-s007.docx]

| Variables | T2DM (n=12) | T2DM/MI (n=15) |
| --- | --- | --- |
| Age (years) | 57 ± 14 | 65 ± 11 |
| Gender (males/female) | 7/5 | 6/15 |
| BMI (kg/m^2^) | 27.8 ± 3.6 | 29.1 ± 5.4 |
| Systolic BP (mmHg) | 131 ± 12 | 131 ± 16 |
| Diastolic BP (mmHg) | 80 ± 10 | 75 ± 12 |
| Fasting glucose (mM) | 13.5 ± 8.2 | 12.4 ± 7.0 |
| HbAlc (%) | 9.3 ± 2.1 | 10.8 ± 3.2 |
| No. of smokers | 6 | 8 |
| Hemoglobin (g/L) | 118 ± 25 | 79 ± 15* |
| Creatinine (μmol/L) | 79 ± 15 | 95 ± 32 |
| Triglycerides (mmol/L) | 2.0 ± 0.8 | 2.0 ± 0.8 |
| Total cholesterol (mmol/L) | 3.6 ± 0.7 | 3.6 ± 0.7 |
| HDL (mmol/L) | 1.0 ± 0.3 | 1.5 ± 0.6 |
| LDL (mmol/L) | 2.0 ± 0.6 | 3.1 ± 0.5* |
| hs-TNT (ng/ml) | 3.4 ± 0.7 | 89.8 ± 26.0*** |
| MYO (μg/ml ) | 56 ± 16 | 1632 ± 231*** |
| CK-MB (μg/ml ) | 3.2 ± 0.8 | 286 ± 23*** |
| Insulin | 8 | 12 |
| Metformin | 12 | 14 |
| GLP-1 analogue | 6 | 6 |
| DPP-4i | 4 | 3 |
| SU | 2 | 2 |
| SGLT2i | 3 | 3 |
| ACEi/ARB | 0 | 0 |
| Aspirin | 0 | 0 |
| Lipid lowering | 10 | 15 |
| β-blocker | 0 | 0 |
| Calcium channel i | 0 | 0 |
| PCI | 0 | 15 |

Table 3. Basic characteristics of T2DM patients complicated with or without MI.

T2DM, type 2 diabetes mellitus; T2DM/MI, type 2 diabetes mellitus complicated with myocardial infarction; BMI, body mass index; HbAlc, Hemoglobin A1c; HDL, high-density lipoprotein; LDL, low-density lipoprotein; hs-cTNT, highly sensitive cardiac troponin T; MYO, Myoglobin; CK-MB, creatine kinase-muscle/brain; GLP-1, glucagon like peptide-1; DPP-4i, dipeptidyl peptidase-4 inhibitor; SU, sulfonylurea; SGLT2i, sodium-glucose co-transporter inhibitor; ACEi, angiotensin-converting enzyme inhibitor; ARB, angiotensin receptor blocker; PCI, Percutaneous coronary intervention. Data are expressed as mean ± SD; * *p* < 0.05, *** *p* < 0.001.
